# ERCC2 polymorphisms and radiation-induced adverse effects on normal tissue: systematic review with meta-analysis and trial sequential analysis

**DOI:** 10.1186/s13014-015-0558-6

**Published:** 2015-12-01

**Authors:** Yu-Zhe Song, Mei-Na Duan, Yu-Yu Zhang, Wei-Yan Shi, Cheng-Cheng Xia, Li-Hua Dong

**Affiliations:** Department of Radiation Oncology, the First Hospital of Jilin University, 71 Xinmin Avenue, Changchun, Jilin China; Department of Respiratory Medicine, the First Hospital of Jilin University, Changchun, Jilin China

**Keywords:** ERCC2, Polymorphism, Radiotherapy, Adverse effect, Radiogenomics

## Abstract

**Background:**

The relationship between ERCC2 polymorphisms and the risk of radiotoxicity remains inconclusive. The aim of our study is to systematically evaluate the association between ERCC2 polymorphisms and the risk of radiotoxicity.

**Methods:**

Publications were identified through a search of the PubMed and Web of Science databases up to August 15, 2015. The pooled odds ratios (ORs) with corresponding 95 % confidence intervals (CIs) were calculated to evaluate the association between ERCC2 polymorphisms and radiotoxicity. Trial sequential analysis (TSA) and power calculation were performed to evaluate the type 1 and type 2 errors.

**Results:**

Eleven studies involving 2584 patients were ultimately included in this meta-analysis. Conventional meta-analysis identified a significant association between ERCC2 rs13181 polymorphism and radiotoxicity (OR = 0.71, 95 % CI: 0.55-0.93, *P* = 0.01), but this association failed to get the confirmation of TSA.

**Conclusions:**

The minor allele of rs13181 polymorphism may confer a protect effect against radiotoxicity. To confirm this correlation at the level of OR = 0.71, an overall information size of approximate 2800 patients were needed.

**Electronic supplementary material:**

The online version of this article (doi:10.1186/s13014-015-0558-6) contains supplementary material, which is available to authorized users.

## Introduction

Radiotherapy is commonly used in cancer treatment. At least half of cancer patients will require radiotherapy with either curative or palliative intent [1]. However, the adverse effect induced by radiotherapy restricts this modality from playing a larger role in the multidisciplinary therapy of cancer. It has been widely noticed that patients were not homogeneous in the reaction of normal tissue following radiotherapy [2, 3]. The standard radiotherapy schedule was recommended treating the cancer patients as a whole, which was actually miscellaneous with patients of different radio-sensitivity. So the radio-resistive patients who can bear more doses of radiotherapy were confined in the protocol of standard radiotherapy with the radio-sensitive patients who may even fail the cost-benefit evaluation of receiving radiotherapy. Consequently, the likelihood of a cure was to some extent reduced for some patients. On the other hand, for some others, the standard radiotherapy was still too harmful to the balance between the therapeutic effect and the normal tissue injury. It is believed that a genetic basis plays an important role in this heterogeneous response to radiation [4, 5].

‘Radiogenomics’ is the study of genetic variation associated with response to radiotherapy, with a main purpose of establishing single nucleotide polymorphism (SNP) based risk models that can stratify patients according to radio-sensitivity [6, 7]. In the last decade, candidate gene association studies have identified several potential predictors for radio-sensitivity. Due to the insufficient sample size of these studies and relatively small effects conferred by relevant SNPs, it makes much sense to systematically synthesize the previous evidence. Recently, we have reviewed several SNPs in radiogenomics, including ATM [8], XRCC3 [9] and XRCC1. The aim of the present meta-analysis is to evaluate the effect of excision repair cross-complementing 2 (ERCC2, also known as XPD, xeroderma pigmentosum group D ) polymorphisms on radiotoxicity.

ERCC2 protein is an essential component of the general transcription factor TFIIH complex that plays a key role in nucleotide excision repair (NER) and basal transcription [10–14]. Besides a 5’-3’ helicase activity, ERCC2 also plays a bridging function within the TFIIH complex [13, 15]. Mutations in ERCC2/XPD have been associated with three hereditary diseases, namely Xeroderma pigmentosum (XP), Cockayne Syndrome and Trichothyodystrophy (TTD) [10, 13, 16]. However, *in vitro* studies failed to relate the polymorphisms of ERCC2 to DNA repair capacity [13, 17–19].

## Materials and methods

### Search strategy and inclusion criteria

Two investigators independently searched the PubMed, and Web of Science databases through a comprehensive search strategy including the terms “SNP”, “ERCC2/XPD”, “radiotherapy” and “toxicity” (see Additional file 1 for the specific strategy). The searching result was last updated on August 15, 2015. Studies satisfying the following criteria were eligible for inclusion: (1) case–control study or cohort study; (2) evaluated the effect of ERCC2 polymorphisms on radiotoxicity; (3) adequate information provided to calculate the odds ratio (OR) and the corresponding 95 % confidence interval (CI). There were no limitations on the language of publication. To avoid exaggerating the representation of certain study, the effect of each study should be cumulated only one time in the synthetical result. If a single study evaluated multiple toxicities, we included the most common toxicity to reduce the potential heterogeneity between studies.

### Data extraction

Two investigators independently extracted data from each included study using a standard data collection form. The following information was extracted: first author’s surname, year of publication, country of origin, ethnicity, cancer type, radiotoxicity, assessment criteria, and ORs with the corresponding 95 % CI for the association between ERCC2 polymorphisms and radiotoxicity. Study authors were contacted when the data provided was insufficient. Disagreements were resolved by discussion among all investigators.

### Statistical methods

The pooled OR and 95 % CI were calculated to assess the strength of the association between ERCC2 polymorphisms and the risk of radiotoxicity. Meta-analysis was first performed on each included polymorphism under 3 genetic comparison models: dominant model (mutant homozygote/heterozygote vs. wild type), recessive model (mutant homozygote vs. wild type/heterozygote) and allelic model (minor allele vs. common allele). Subsequently, subgroup analyses were conducted by adverse effect. The heterogeneity between studies was assessed with the chi-squared based Q-test and *I*^2^ statistics [20, 21]. When the chi-squared *P* was <0.10 or the *I*^2^ statistic was ≥50 %, the heterogeneity was considered statistically significant, and a random-effects model (DerSimonian-Laird method) was applied [22]; otherwise, a fixed-effects model (Mantel-Haenszel method) was accepted [23]. Sensitivity analysis was performed to evaluate the stability and reliability of the pooled results by excluding each study individually and reanalyzing the remaining studies. Publication bias was evaluated via Begg’s funnel plot and Egger’s test [24, 25]. If publication bias existed, the “trim and fill” method was applied to estimate the number of missing studies and to adjust the pooled result [26]. Statistical power calculation was performed to evaluate the potential type 2 errors for the primary and subgroup analyses. A two-sided *P* <0.05 was considered significant for all the analyses except the heterogeneity tests. Meta-analyses were performed using Stata (Version 14.0, StataCorp LP, College Station, TX, USA).

Trial sequential analysis (TSA) was performed for the analyses involving more than six studies. A two sided α = 0.05, β = 0.2 and a relative risk reduction (RRR) of 10 % were used. A required information size was estimated with the adjustment by diversity (*D*^2^) between studies [27, 28]. An α-spending boundary and a futility boundary were constructed accordingly. The cumulative z-curve was produced by plotting a series of z-values of cumulative meta-analyses [29]. If the accrued number of included patients surpassed the required information size, it means that a sufficient level of evidence has been reached. If the curve crossed the α-spending boundary, it was considered that the conclusion of significant association was confirmed. When the z-curve crossed the futility boundary, the conclusion of indiscrimination between two groups was accepted under the given conditions [30]. TSA was performed using trial sequential analysis software version 0.9 beta [29].

## Results

### Eligible studies

**Fig. 1:**
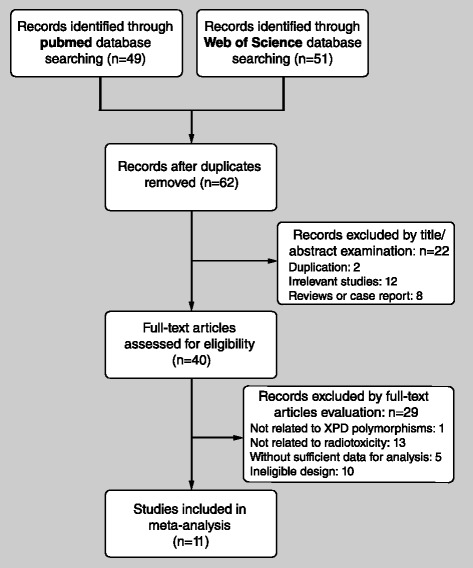
Flow diagram of the literature review process

A flow diagram summarizing the literature review process and reasons for exclusion is presented in Fig. 1. A total of 11 studies involving 502 cases and 2082 controls were eventually included in this meta-analysis. The baseline characteristics of the included studies are presented in Table 1. The studies were published from 2005 to 2013, and the sample sizes ranged from 60 to 698. Three SNPs of ERCC2 were involved in the present meta-analysis: ERCC2 Lys751Gln (rs13181) (nine studies), Asp312Asn (rs1799793) (five studies) and Asp711 (rs1052555) (two studies). Cancer categories included breast cancer (six studies), lung cancer (one study), esophageal cancer (one study), prostatic carcinoma (one study), bladder cancer (one study) and rectal cancer (one study).

**Table 1:** Baseline Characteristics of the Eligible Studies

Author, Year	Country	Ethnicity	Disease	Adverse Effect	Assessment Criteria	SNP	MAF	Sample Size	EBRT Dose, Gy
Chang-Claude 2005 [40]	Germany	Caucasian	Breast cancer	Early effect: acute skin toxicity	RTOG/EORTC ≥ G2	rs13181, rs1799793	0.378,0.368	441	50-60
Chang-Claude 2009 [41]	Germany	Caucasian	Breast cancer	Late effect: telangiectasia	RTOG/EORTC ≥ G2	rs1799793	0.363	400	50-60
Duldulao 2013 [42]	USA	NR	Rectal cancer	Early effect: gastrointestinal and abdominal toxicity	CTCAE v3.0 ≥ G3	rs13181	0.276	156	50.4-54
Fachal 2012 [43]	Spain	Caucasian	Prostate cancer	Early effect: gastrointestinal toxicity	CTCAE v2.0 ≥ G2	rs1799793, rs1052555	0.342,0.328	698	70-76
Mangoni 2011 [44]	Italy	Caucasian	Breast cancer	Early effect: acute skin toxicity	CTCAE v2.0 ≥ G2c^a^	rs13181, rs1799793	NA	87	50-62.8
Raabe 2012 [45]	Germany	NR	Breast cancer	Early effect: acute skin toxicity	RTOG/EORTC ≥ G2	rs13181	0.646	82	50.4
Sakano 2010 [46]	Japan	Asian	Bladder cancer	Early effect: diarrhea	CTCAE v3.0 ≥ G2	rs13181	NA	93	30.0-60.4
Terrazzino 2012 [47]	Italy	Caucasian	Breast cancer	Early effect: acute skin toxicity	RTOG/EORTC ≥ G2	rs13181	0.365	285	50-66
Yoon 2011 [48]	USA	Mixed^b^	Esophageal cancer	Early effect: esophageal toxicity and myelosuppression	RTOG/EORTC ≥ G3	rs13181, rs1799793, rs1052555	NA	60	46
Zhang 2010 [49]	China	NR	Lung cancer	Early effect: esophageal toxicity	NR v2.0 ≥ G2	rs13181	0.286	213	50-70
Zschenker 2010 [50]	Germany	NR	Breast cancer	Late effect: fibrosis	LENT/SOMA ≥ G2	rs13181	0.551	69	54-55

*Abbreviations*: *MAF* minor allele frequency, *RTOG* the radiation therapy oncology group, *EORTC* European Organization for Research and Treatment of Cancer, *EBRT* external beam radiation therapy, *CTCAE* Common Terminology Criteria for Adverse Events. *NR* not related. *NA* not available. *SNP* single nucleotide polymorphism

^a^method based on CTCAE, in which G2c was defined as at least one moist desquamation or interruption of radiotherapy due to toxicity. ^b^Caucasian account for 93 %

### Meta-analysis results

**Fig. 2:**
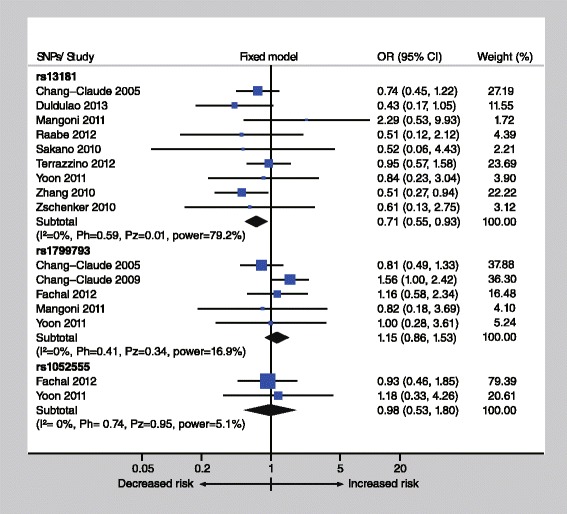
Meta-analysis between ERCC2 polymorphisms and risk of radiotoxicity. A fixed-effects model was used. The square with the corresponding horizontal line represents the OR and 95 % CI of each study. The area of the square reflects the weight of the study. The diamond represents the pooled OR and 95 % CI. Power listed in the graph was calculated at the level of the corresponding OR

**Fig. 3:**
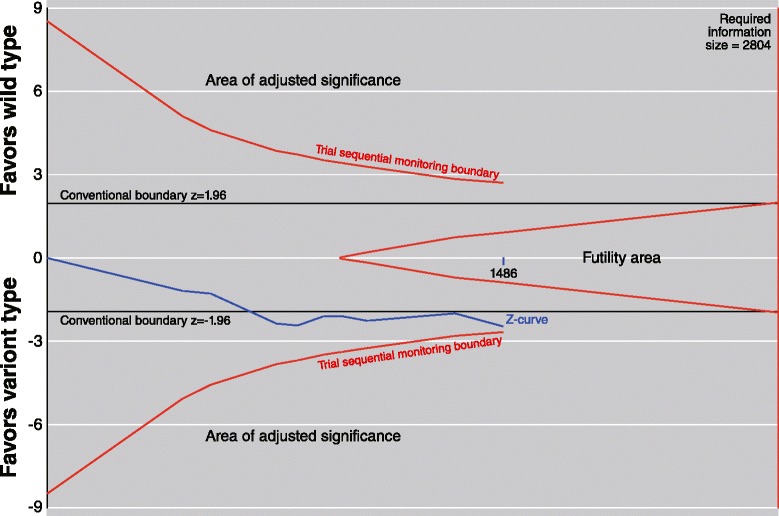
Trial sequential analysis for rs13181 polymorphism on overall radiotoxicity. A diversity (D^2^) adjusted information size was calculated using α = 0.05 (two-sided), β = 0.20 (power 80 %), and an anticipated relative risk reduction of 10 %. Diversity and control event proportion were set basing the actual status of included data. The cumulative z-curve crossed the conventional boundary for significance, but did not cross any adjusted boundary, leaving the meta-analysis inconclusive of an effect of RRR = 10 %

Significant associations between rs13181 and risk of radiotoxicity were identified in dominant model (OR = 0.71, 95 % CI: 0.55-0.93, *P* = 0.01) and allelic model (OR = 0.78, 95 % CI: 0.64-0.97, *P* = 0.02) by conventional meta-analysis (Fig. 2, Table 2). TSA suggested that more than half (1486/2804) of the required information size has been accrued (Fig. 3). The cumulative z-curve (dominant model) has transcended the conventional significance boundary, which was in accordance with the result of conventional meta-analysis. No trial sequential monitoring boundary was further crossed, leaving the meta-analysis inconclusive of a 10 % relative risk reduction. For rs1799793 and rs1052555 polymorphisms, no significant association was identified.

**Table 2:** Summary of meta-analysis results for the association between ERCC2 polymorphisms and radiotoxicity

Variable	No. of cohorts	Cases/Total	OR [95 % CI]^a^	*P*_z-test_	*I* ^2^ (%)	*P*_het_	Power (%)
rs13181							
Dominant model	9	342/1486	0.71 [0.55, 0.93]	0.01	0	0.59	79.2
Early adverse effect	8	325/1417	0.72 [0.55, 0.94]	0.02	0	0.49	73.8
Acute skin toxicity	4	219/895	0.85 [0.61, 1.20]	0.36	0	0.43	18.0
Acute esophageal toxicity	2	70/273	0.56 [0.32, 0.97]	0.04	0	0.49	54.6
Acute gastrointestinal toxicity	2	36/249	0.44 [0.19, 1.02]	0.06	0	0.87	52.7
Recessive model	5	286/1090	0.75 [0.50, 1.12]	0.16	43	0.14	27.4
Allelic model	5	286/1090	0.78 [0.64, 0.97]	0.02	27	0.24	68.5
rs1799793							
Dominant model	5	256/1687	1.15 [0.86, 1.53]	0.34	0	0.41	16.9
Early adverse effect	4	130/1287	1.36 [0.96, 1.92]	0.09	0	0.75	35.2
Acute skin toxicity	2	84/529	1.48 [0.97, 2.26]	0.07	0	0.42	31.9
Recessive model	3	236/1540	0.67 [0.41, 1.08]	0.10	0	0.49	38.9
Allelic model	3	236/1540	0.99 [0.80, 1.23]	0.95	44	0.17	5.1
rs1052555							
Dominant model	2	46/758	0.98 [0.53, 1.80]	0.95	0	0.74	5.1

*P*_Z-test_: *P* value of Z-Test for overall effect

*P*_het_: *P* value of Chi^2^ based Q-test for heterogeneity

^a^Fixed-effect model used

Subgroup analyses were performed only under dominant model, due to only five studies were included under recessive model and allelic model. A significant association between rs13181 and esophageal toxicity was identified by subgroup analysis by adverse effect (OR = 0.71, 95 % CI: 0.55-0.93, *P* = 0.01). No significant association was found with acute skin toxicity (OR = 0.85, 95 % CI: 0.61-1.20, *P* = 0.36) and gastrointestinal toxicity (OR = 0.44, 95 % CI: 0.19-1.02, *P* = 0.06) (Table 2). Due to most included studies of rs13181 (1417/1486 patients) evaluated early adverse effect, the pooled result of early adverse effect approximates with the result of overall toxicity (OR = 0.72, 95 % CI: 0.55-0.94, *P* = 0.02) (Table 2).

### Heterogeneity and sensitivity analyses

**Fig. 4:**
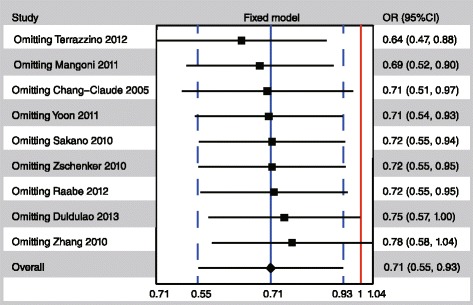
Sensitivity analysis for rs13181 polymorphism

The heterogeneities between studies of all analyses were not significant. The pooled results present stable in the sensitivity analysis (Fig. 4). The study Terrazzino 2012 possesses the greatest influence to the direction favors wild type, while Zhang 2010 owns the greatest influence to the direction of favors variant type. After omitting the study Zhang 2010, the pooled result was changed into non-significance (OR = 0.78, 95 % CI: 0.58-1.04, *P* = 0.1).

### Publication bias

The distribution of all the included studies of rs13181 in Begg’s funnel plot was visually symmetrical (Additional file 1: Figure S1), and the *p*-value of egger’s test was 0.891, which indicated that no obvious publication bias exists in our meta-analysis of rs13181.

## Discussion

Radiogenomics has entered the era of big data [31]. However, for the last decade candidate gene approach was predominant, and inconsistent results have been reported due to most studies were underpowered with a relatively small information size. Besides, some single SNP may confer only slightly elevated risk of radiotoxicity, so it is difficult to identify this true effect without an enough sample size. Hence, systematically summarizing the previous data benefits of identifying a relatively small but significant effect of relevant SNPs. In fact, meta-analysis has played an important role in radiogenomics.

The present meta-analysis systematically summarized the previous data of ERCC2 in radiogenomics. A significant association between rs13181 and radiotoxicity was identified by conventional meta-analysis. Our data revealed that the major allele of rs13181 presents as a risk allele, which means the minor allele confers a protect effect against the appearance of radiotoxicity. However, we should notice that this association was still borderline (95 % CI: 0.55-0.93), and one study made the original significance vanish in leave-one-out sensitivity analysis. In addition, this conventional significance failed to get the confirmation of TSA. We applied TSA with the intention of drawing out more specific conclusions. TSA revealed that the z-curve failed to further cross the α-spending boundary after crossing the conventional boundary of z = −1.96, which means the correlation between rs13181 polymorphism and radiotoxicity risk still need the confirmation of subsequent studies. To safely conclude an effect of OR = 0.71, additional 1400 patients were needed.

We performed subgroup analyses by adverse effect. Radiation-induced adverse effects can be classified as early or late effects according to the time before the manifestation of relevant clinical symptoms. Most radiogenomics studies reported early and late effects separately, and some SNPs exert inconformity effect on early and late effects. For instance, the pooled data of XRCC1 revealed that Arg399Gln (rs25487) polymorphism significantly correlated with an elevated risk of early radiotoxicity, while this SNP was ruled out any clinical relevance with late radiotoxicity (Song YZ: The XRCC1 Arg399Gln Polymorphism and Radiation-Induced Adverse Effects on Normal Tissue: Systematic Review with Meta-analysis and Trial Sequential Analysis. Submitted). Meta-analysis of XRCC3 Thr241Met (rs861539) polymorphism also reported a similar result, that is a significant association with early radiotoxicity rather than late radiotoxicity [9]. In the present meta-analysis, most studies evaluated early radiotoxicity, and only one study involved late radiotoxicity, so it is noteworthy that the pooled result of the present meta-analysis mainly reflects the effect on early radiotoxicity. Within early radiotoxicity, a significant association between rs13181 polymorphism and acute esophageal toxicity was also identified. However, this association should not be over interpreted, due to only two studies were included in this subgroup. The most evaluated radiotoxicity was acute skin toxicity in the present meta-analysis, and four studies evaluated this reaction following radiotherapy. The association between rs13181 polymorphism and acute skin toxicity was not significant.

For interpreting the pooled result of a meta-analysis, adequate evaluation on the heterogeneity between studies is crucial (as was previously described in detail [9]). One of the most important source of heterogeneity derived from the heterogeneous treatment protocols among the included studies. For instance, the radiotherapy parameters, such as total dose, dose per fraction and irradiation volume, were not identical among the included studies. Some treatment protocols of included studies involved chemotherapy as a component of multidisciplinary therapy, while some were basing on radiotherapy alone. Toxicity evaluation was another important source of heterogeneity. Both the criteria applied and the division grade were not consistent among included studies. Nevertheless, statistical calculations did not identify obvious heterogeneity, we believed that the influence of these potential heterogeneity factors were at an acceptable level.

Based on the radiogenomics studies throughout the last decade, it is safe to conclude that no SNP alone possesses the power to accurately predict the radio-sensitivity prior radiotherapy [6, 31]. A study aiming to validate the associations previously reported between candidate SNPs and radiotoxicity did not confirm any significant association [32]. To date, six genome-wide association studies (GWASs) have been published on radiogenomics [33–38]. The SNPs which were identified with a genome-wide significance were not located in the region supposed by candidate gene association studies. However, statically significant conclusions were constantly reported by meta-analyses of candidate gene association studies. Through combining information of all the relevant studies, more statistical power was acquired [39]. Evidence of meta-analysis has revealed that XRCC1 rs25487, XRCC3 rs861539, ATM rs1801516 and ERCC2 rs13181 polymorphisms significantly associated with early radiotoxicity, though the effect size was relatively small [8, 9]. While an individual candidate SNP was not expected to confer a large effect on radiotoxicity. Instead, composing a synthesized risk model is the major modality how the relevant SNPs play a role on the prediction of radio-sensitivity. Despite a relatively small effect (with odds radios of 1.2 to 1.5) exerted on radiotoxicity by individual SNP, an enough prediction power can be accrued by involving multiple such SNPs.

## Conclusions

Although the minor allele of rs13181 polymorphism was identified with a protect effect against radiotoxicity, it is noteworthy that the correlation was borderline, and one included study made the overall meta-analysis loss the statistical significance in leave-one-out sensitivity analysis. In addition, this significant association identified by traditional meta-analysis failed to get the conformation of TSA. More studies with additional 1400 patients were needed to draw the firm conclusion at the level of OR = 0.71.

## Additional file

Additional file 1: Figure S1. Begg’s funnel plot for the meta-analysis of rs13181. Supplementary material: The specific search strategy. (PDF 305 kb)

## Abbreviations

CIs: confidence intervals
D2: diversity
ED: erectile dysfunction
ERCC2: excision repair cross-complementing 2
MAF: minor allele frequency
NER: nucleotide excision repair
ORs: odds ratios
RRR: relative risk reduction
SNP: single nucleotide polymorphism
TSA: trial sequential analysis
TTD: Trichothyodystrophy
XP: xeroderma pigmentosum
XPD: xeroderma pigmentosum group D

## References

[1] Burnet NG, Elliott RM, Dunning A, West CM (2006). Radiosensitivity, radiogenomics and RAPPER. Clin Oncol (R Coll Radiol).

[2] Bentzen SM, Overgaard J (1994). Patient-to-patient variability in the expression of radiation-induced normal tissue injury. Semin Radiat Oncol.

[3] Henriquez-Hernandez LA, Bordon E, Pinar B, Lloret M, Rodriguez-Gallego C, Lara PC (2012). Prediction of normal tissue toxicity as part of the individualized treatment with radiotherapy in oncology patients. Surg Oncol.

[4] Andreassen CN, Alsner J, Overgaard J (2002). Does variability in normal tissue reactions after radiotherapy have a genetic basis—where and how to look for it?. Radiother Oncol.

[5] Ho AY, Atencio DP, Peters S, Stock RG, Formenti SC, Cesaretti JA (2006). Genetic predictors of adverse radiotherapy effects: the Gene-PARE project. Int J Radiat Oncol Biol Phys.

[6] Kerns SL, Ostrer H, Rosenstein BS (2014). Radiogenomics: using genetics to identify cancer patients at risk for development of adverse effects following radiotherapy. Cancer Discov.

[7] Kerns SL, West CM, Andreassen CN, Barnett GC, Bentzen SM, Burnet NG (2014). Radiogenomics: the search for genetic predictors of radiotherapy response. Future Oncol.

[8] Dong L, Cui J, Tang F, Cong X, Han F (2015). Ataxia telangiectasia-mutated gene polymorphisms and acute normal tissue injuries in cancer patients after radiation therapy: a systematic review and meta-analysis. Int J Radiat Oncol Biol Phys.

[9] Song YZ, Han FJ, Liu M, Xia CC, Shi WY, Dong LH (2015). Association between single nucleotide polymorphisms in XRCC3 and radiation-induced adverse effects on normal tissue: a meta-analysis. PLoS One.

[10] Vashisht AA, Yu CC, Sharma T, Ro K, Wohlschlegel JA (2015). The association of the Xeroderma Pigmentosum Group D DNA helicase (XPD) with transcription factor IIH is regulated by the cytosolic iron-sulfur cluster assembly pathway. J Biol Chem.

[11] Liu H, Rudolf J, Johnson KA, McMahon SA, Oke M, Carter L (2008). Structure of the DNA repair helicase XPD. Cell.

[12] Liu Z, Zhao W, Zhang Q, Lai L, Jiang S, Zhang J, et al. Increased Oxidative Damage and Reduced DNA Repair Enzyme XPD Involvement in High Glucose-Mediated Enhancement of Levobupivacaine-Induced Neurotoxicity. Neurochem Res. 2015. doi: 10.1007/s11064-015-1685-z. PubMed 10.1007/s11064-015-1685-z 26264262

[13] Laine JP, Mocquet V, Bonfanti M, Braun C, Egly JM, Brousset P (2007). Common XPD (ERCC2) polymorphisms have no measurable effect on nucleotide excision repair and basal transcription. DNA Repair (Amst).

[14] Drapkin R, Reardon JT, Ansari A, Huang JC, Zawel L, Ahn K (1994). Dual role of TFIIH in DNA excision repair and in transcription by RNA polymerase II. Nature.

[15] Reardon JT, Ge H, Gibbs E, Sancar A, Hurwitz J, Pan ZQ (1996). Isolation and characterization of two human transcription factor IIH (TFIIH)-related complexes: ERCC2/CAK and TFIIH. Proc Natl Acad Sci U S A.

[16] Lehmann AR (2001). The xeroderma pigmentosum group D (XPD) gene: one gene, two functions, three diseases. Genes Dev.

[17] Qiao Y, Spitz MR, Shen H, Guo Z, Shete S, Hedayati M (2002). Modulation of repair of ultraviolet damage in the host-cell reactivation assay by polymorphic XPC and XPD/ERCC2 genotypes. Carcinogenesis.

[18] Spitz MR, Wu X, Wang Y, Wang LE, Shete S, Amos CI (2001). Modulation of nucleotide excision repair capacity by XPD polymorphisms in lung cancer patients. Cancer Res.

[19] Duell EJ, Wiencke JK, Cheng TJ, Varkonyi A, Zuo ZF, Ashok TD (2000). Polymorphisms in the DNA repair genes XRCC1 and ERCC2 and biomarkers of DNA damage in human blood mononuclear cells. Carcinogenesis.

[20] Higgins JP, Thompson SG (2002). Quantifying heterogeneity in a meta-analysis. Stat Med.

[21] Higgins JP, Thompson SG, Deeks JJ, Altman DG (2003). Measuring inconsistency in meta-analyses. BMJ.

[22] DerSimonian R, Laird N (1986). Meta-analysis in clinical trials. Control Clin Trials.

[23] Mantel N, Haenszel W (1959). Statistical aspects of the analysis of data from retrospective studies of disease. J Natl Cancer Inst.

[24] Begg CB, Mazumdar M (1994). Operating characteristics of a rank correlation test for publication bias. Biometrics.

[25] Egger M, Davey Smith G, Schneider M, Minder C (1997). Bias in meta-analysis detected by a simple, graphical test. BMJ.

[26] Duval S, Tweedie R (2000). Trim and fill: a simple funnel-plot-based method of testing and adjusting for publication bias in meta-analysis. Biometrics.

[27] Wetterslev J, Thorlund K, Brok J, Gluud C (2008). Trial sequential analysis may establish when firm evidence is reached in cumulative meta-analysis. J Clin Epidemiol.

[28] Wetterslev J, Thorlund K, Brok J, Gluud C (2009). Estimating required information size by quantifying diversity in random-effects model meta-analyses. BMC Med Res Methodol.

[29] Thorlund K EJ, Wetterslev J, Brok J, Imberger G, Gluud C. User manual for trial sequential analysis (TSA). Copenhagen Trial Unit, Centre for Clinical Intervention Research, 2011.

[30] Brok J, Thorlund K, Gluud C, Wetterslev J (2008). Trial sequential analysis reveals insufficient information size and potentially false positive results in many meta-analyses. J Clin Epidemiol.

[31] Rosenstein BS, West CM, Bentzen SM, Alsner J, Andreassen CN, Azria D (2014). Radiogenomics: radiobiology enters the era of big data and team science. Int J Radiat Oncol Biol Phys.

[32] Barnett GC, Coles CE, Elliott RM, Baynes C, Luccarini C, Conroy D (2012). Independent validation of genes and polymorphisms reported to be associated with radiation toxicity: a prospective analysis study. Lancet Oncol.

[33] Kerns SL, Ostrer H, Stock R, Li W, Moore J, Pearlman A (2010). Genome-wide association study to identify single nucleotide polymorphisms (SNPs) associated with the development of erectile dysfunction in African-American men after radiotherapy for prostate cancer. Int J Radiat Oncol Biol Phys.

[34] Kerns SL, Stone NN, Stock RG, Rath L, Ostrer H, Rosenstein BS (2013). A 2-stage genome-wide association study to identify single nucleotide polymorphisms associated with development of urinary symptoms after radiotherapy for prostate cancer. J Urol.

[35] Kerns SL, Stock RG, Stone NN, Blacksburg SR, Rath L, Vega A (2013). Genome-wide association study identifies a region on chromosome 11q14.3 associated with late rectal bleeding following radiation therapy for prostate cancer. Radiother Oncol.

[36] Kerns SL, Stock R, Stone N, Buckstein M, Shao Y, Campbell C (2013). A 2-stage genome-wide association study to identify single nucleotide polymorphisms associated with development of erectile dysfunction following radiation therapy for prostate cancer. Int J Radiat Oncol Biol Phys.

[37] Fachal L, Gómez-Caamaño A, Barnett GC, Peleteiro P, Carballo AM, Calvo-Crespo P, et al. A three-stage genome-wide association study identifies a susceptibility locus for late radiotherapy toxicity at 2q24.1. Nature genetics. 2014;46(8):891-4. Epub 2014/07/01. doi: 10.1038/ng.3020. PubMed PMID: 24974847. 10.1038/ng.3020 24974847

[38] Barnett GC, Thompson D, Fachal L, Kerns S, Talbot C, Elliott RM (2014). A genome wide association study (GWAS) providing evidence of an association between common genetic variants and late radiotherapy toxicity A three-stage genome-wide association study identifies a susceptibility locus for late radiotherapy toxicity at 2q24.1. Radiother Oncol.

[39] Higgins JPT, Green S. Cochrane Handbook for systematic reviews of interventions, version 5.0.0. John Wiley & Sons, 2009.

[40] Chang-Claude J, Popanda O, Tan XL, Kropp S, Helmbold I, von Fournier D (2005). Association between polymorphisms in the DNA repair genes, XRCC1, APE1, and XPD and acute side effects of radiotherapy in breast cancer patients. Clin Cancer Res.

[41] Chang-Claude J, Ambrosone CB, Lilla C, Kropp S, Helmbold I, von Fournier D (2009). Genetic polymorphisms in DNA repair and damage response genes and late normal tissue complications of radiotherapy for breast cancer. Br J Cancer.

[42] Duldulao MP, Lee W, Nelson RA, Ho J, Le M, Chen Z (2013). Gene polymorphisms predict toxicity to neoadjuvant therapy in patients with rectal cancer. Cancer.

[43] Fachal L, Gomez-Caamano A, Peleteiro P, Carballo A, Calvo-Crespo P, Sanchez-Garcia M (2012). Association of a XRCC3 polymorphism and rectum mean dose with the risk of acute radio-induced gastrointestinal toxicity in prostate cancer patients. Radiother Oncol.

[44] Mangoni M, Bisanzi S, Carozzi F, Sani C, Biti G, Livi L (2011). Association between genetic polymorphisms in the XRCC1, XRCC3, XPD, GSTM1, GSTT1, MSH2, MLH1, MSH3, and MGMT genes and radiosensitivity in breast cancer patients. Int J Radiat Oncol Biol Phys.

[45] Raabe A, Derda K, Reuther S, Szymczak S, Borgmann K, Hoeller U (2012). Association of single nucleotide polymorphisms in the genes ATM, GSTP1, SOD2, TGFB1, XPD and XRCC1 with risk of severe erythema after breast conserving radiotherapy. Radiat Oncol.

[46] Sakano S, Hinoda Y, Sasaki M, Wada T, Matsumoto H, Eguchi S (2010). Nucleotide excision repair gene polymorphisms may predict acute toxicity in patients treated with chemoradiotherapy for bladder cancer. Pharmacogenomics.

[47] Terrazzino S, La Mattina P, Masini L, Caltavuturo T, Gambaro G, Canonico PL (2012). Common variants of eNOS and XRCC1 genes may predict acute skin toxicity in breast cancer patients receiving radiotherapy after breast conserving surgery. Radiother Oncol.

[48] Yoon HH, Catalano P, Gibson MK, Skaar TC, Philips S, Montgomery EA (2011). Genetic variation in radiation and platinum pathways predicts severe acute radiation toxicity in patients with esophageal adenocarcinoma treated with cisplatin-based preoperative radiochemotherapy: results from the Eastern Cooperative Oncology Group. Cancer Chemother Pharmacol.

[49] Zhang L, Yang M, Bi N, Ji W, Wu C, Tan W (2010). Association of TGF-beta1 and XPD polymorphisms with severe acute radiation-induced esophageal toxicity in locally advanced lung cancer patients treated with radiotherapy. Radiother Oncol.

[50] Zschenker O, Raabe A, Boeckelmann IK, Borstelmann S, Szymczak S, Wellek S (2010). Association of single nucleotide polymorphisms in ATM, GSTP1, SOD2, TGFB1, XPD and XRCC1 with clinical and cellular radiosensitivity. Radiother Oncol.

