# Supplementary material for: ERCC2 polymorphisms and radiation-induced adverse effects on normal tissue: systematic review with meta-analysis and trial sequential analysis
Source: Radiat Oncol. 2015 Dec 1;10:247. doi: 10.1186/s13014-015-0558-6 (PMC4665885; doi:10.1186/s13014-015-0558-6)
Supplement: Supplementary file 1 — Begg’s funnel plot for the meta-analysis of rs13181. Supplementary material: The specific search strategy. (PDF 305 kb) [file 13014_2015_558_MOESM1_ESM.pdf]

RADIATION ONCOLOGY

Supplementary appendix

Supplement to: ERCC2 Polymorphisms and Radiation-Induced Adverse Effects on Normal Tissue: Systematic Review with Meta-analysis and Trial Sequential Analysis

Supplementary Fig. 1. Begg’s funnel plot for the meta-analysis of rs13181.

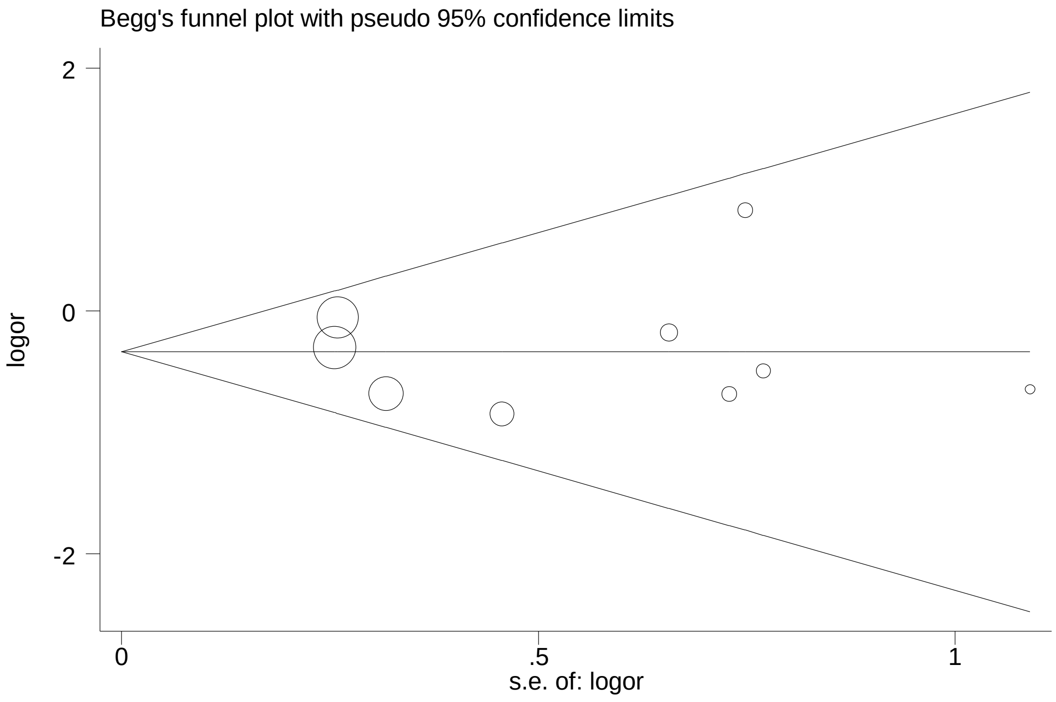

The specific search strategy:

#1 radiotherapy

#2 radiation therapy

#3 radiation

#4 #1 OR #2 OR #3

#5 injury

#6 injuries

#7 toxicity

#8 toxicities

#9 complication

#10 complications

#11 adverse effect

#12 adverse effects

#13 side effect

#14 side effects

#15 #5 OR #6 OR #7 OR #8 OR #9 OR #10 OR #11 OR #12 OR #13 OR #14

#16 XPD OR "xeroderma pigmentosum group D" OR ERCC2 OR "excision repair cross-complementing 2" OR "excision repair cross-complementing group 2")

#17 "xeroderma pigmentosum group D"

#18 ERCC2

#19 "excision repair cross-complementing group 2"

#20 #16 OR #17 OR #18 OR #19

#21 Polymorphism

#22 variant

#23 variation

#24 mutant

#25 genotype

#26 SNPs

#27 single nucleotide polymorphisms

#28 adverse effect

#29 #21 OR #22 OR #23 OR #24 OR #25 OR #26 OR #27 OR #28

#30 #4 AND #15 AND #20 AND #29
